# *Brucella* Omp25 activates the unfolded protein response to promote intracellular proliferation and inflammation

**DOI:** 10.1016/j.jbc.2026.111333

**Published:** 2026-02-26

**Authors:** Jin-Ke Yang, Shuang Huang, Yuan-Pan Hou, Hui-Fei Yuan, Yue Wang, Mi Li, Li-Bo Cao, Tian Xia, Hong-Bing Shu, Xin Wu

**Affiliations:** 1 State Key Laboratory of Animal Disease Control and Prevention, Lanzhou Veterinary Research Institute, Chinese Academy of Agricultural Sciences, Lanzhou, China; 2 Gansu Province Research Center for Basic Disciplines of Pathogen Biology, Lanzhou, China; 3 College of Veterinary Medicine, Lanzhou University, Lanzhou, China; 4 Department of Infectious Diseases, Medical Research Institute, Taikang Center for Life and Medical Sciences, Zhongnan Hospital of Wuhan University, Wuhan University, Wuhan, China

**Keywords:** *Brucella*, Omp25, unfolded protein response, BiP, inflammation

## Abstract

Brucellosis is a widespread zoonotic disease caused by *Brucella*, a genus of facultative intracellular bacteria that infects livestock and humans. *Brucella* primarily replicates within the endoplasmic reticulum (ER) of host cells, where it establishes a specialized replicative niche. This ER localization disrupts ER structure and induces ER stress. The unfolded protein response (UPR) is a critical cellular pathway that maintains ER homeostasis by restoring protein-folding capacity and regulating stress responses. However, how *Brucella* manipulates host UPR pathways to promote its intracellular survival and pathogenesis remains poorly understood. Here, we identify the *Brucella* outer membrane protein Omp25 as a key factor in promoting its intracellular survival and proliferation by activating the host UPR. Omp25 directly binds to the ER chaperone binding-immunoglobulin protein, inducing the release and activation of the UPR sensors, PKR-like ER kinase, inositol-requiring enzyme 1 alpha, and activating transcription factor 6, thereby modulating ER homeostasis to favor bacterial replication. In addition, Omp25 enhances inflammatory cytokine expression *via* the binding-immunoglobulin protein–inositol-requiring enzyme 1 alpha–NF-κB signaling axis. The *omp25*-deleted strains (Δ*omp25*) show impaired intracellular replication and reduced UPR activation and result in attenuated induction of inflammatory genes in infected cells compared with WT strains. *In vivo*, mice infected with an *omp25* mutant strain exhibit lower bacterial burdens and milder tissue pathology compared with mice infected with the WT strain. These findings uncover a mechanism by which Omp25 facilitates *Brucella* intracellular proliferation through UPR modulation and highlight Omp25 as a potential target for therapeutic interventions and next-generation attenuated vaccines.

Brucellosis, caused by the intracellular Gram-negative bacterium *Brucella*, remains one of the most widespread and economically devastating zoonoses worldwide. It poses a major threat to public health security, food safety, and sustainable economic development, particularly in regions reliant on livestock farming (1, 2). In humans, *Brucella* infection manifests as undulant fever, arthritis, spondylitis, meningitis, and chronic debilitating conditions that significantly reduce quality of life and work capacity. In livestock, such as cattle, sheep, and goats, brucellosis leads to reproductive failures, including abortion, stillbirth, and infertility, inflicting heavy economic losses and perpetuating transmission through contaminated animal products (3, 4). Thus, effective control of brucellosis is not only critical for protecting human health but also vital for safeguarding global agriculture and trade.

The intracellular life cycle of *Brucella* is central to its pathogenicity. Following the invasion of host phagocytic cells, *Brucella* traffics through the endosomal pathway and establishes a replicative niche within the endoplasmic reticulum (ER), forming replicative *Brucella*-containing vacuoles (rBCVs) (5). rBCVs are derived from ER membranes and acquire multiple ER-resident proteins, reflecting their extensive integration into the ER network. Three-dimensional correlative electron microscopy studies further demonstrate structural continuity between rBCVs and the ER, indicating that rBCVs are functionally connected to the ER lumen (6). Within this ER-derived niche, *Brucella* resides in close physical proximity to the ER membrane and luminal environment. Because *Brucella* outer membrane proteins (Omps) are exposed on the bacterial surface, their localization within rBCVs places them in a spatially favorable position to interact with ER-associated host factors. This ER tropism enables *Brucella* to exploit the ER’s rich resources while avoiding lysosomal degradation and immune detection (7, 8). However, such remodeling of ER architecture perturbs ER homeostasis and elicits ER stress, which in turn activates a cellular adaptive program known as the unfolded protein response (UPR) to restore normal ER function (9, 10). Accumulating evidence highlights the ER as a critical site for *Brucella* replication, persistence, and establishment of chronic infection; therefore, it is a critical organelle for *Brucella*–host cell interaction.

The ER is indispensable for cellular physiology, orchestrating protein synthesis and folding, calcium storage, and lipid biosynthesis (11). When misfolded or unfolded proteins accumulate within the ER lumen, the chaperone binding immunoglobulin protein (BiP) dissociates from three transmembrane sensors: PERK (PKR-like ER kinase), IRE1α (inositol-requiring enzyme 1 alpha), and ATF6 (activating transcription factor 6) (12). Upon release from BiP, PERK subsequently undergoes dimerization and autophosphorylation. Activated PERK then phosphorylates eukaryotic initiation factor 2α (eIF2α) to attenuate global protein synthesis while selectively upregulating stress-responsive genes (13). IRE1α is an ER-resident transmembrane protein containing both serine/threonine kinase and endoribonuclease (RNase) domains. Upon activation, IRE1α oligomerizes and undergoes autophosphorylation (14). The activated RNase domain splices XBP1 (X-box binding protein 1) mRNA in the cytoplasm to produce the transcription factor XBP1s (the spliced form of XBP1), which translocates to the nucleus and promotes expression of ER chaperones and other components critical for protein folding. IRE1α can also activate NF-κB, linking the UPR to inflammatory signaling (15, 16). Meanwhile, ATF6 is transported to the Golgi, where it undergoes proteolytic processing to release the active fragment ATF6 (N), which enters the nucleus to promote transcription of genes involved in ER quality control (17). Although the primary role of the UPR is to restore ER homeostasis, prolonged or dysregulated activation can profoundly affect inflammatory pathways and cell fate decisions.

Many pathogens, including bacteria and viruses, have evolved strategies to manipulate the UPR to create intracellular environments conducive to their replication (18). Understanding how pathogens interface with the ER and exploit UPR pathways is therefore critical for elucidating their pathogenic mechanisms. In *Brucella*, several studies have highlighted interactions between bacterial virulence factors and the host UPR machinery. For example, type IV secretion system effector VceC localizes to the ER and alters its structure and results in the induction of an inflammatory response associated with placental pathology, whereas TcpB remodels microtubules and ER structure to support *Brucella* replication (19, 20). However, whether major structural components of *Brucella*, particularly Omps that are positioned at the host–pathogen interface within rBCVs, actively modulate UPR signaling remains largely unexplored. Omps are integral structural components of *Brucella* and play central roles in virulence, immune modulation, and intracellular survival. Among them, Omp25 has been shown to contribute to *in vivo* persistence and virulence, in part through suppression of Toll-like receptor–mediated inflammatory signaling and modulation of host immune responses (21, 22, 23). Given its surface exposure and localization within ER-derived rBCVs, Omp25 is well positioned to influence ER-associated signaling pathways. Nevertheless, whether Omp25 directly engages the UPR machinery to promote *Brucella* intracellular survival and chronic infection has not been determined.

In this study, we identify *Brucella abortus* (*B. abortus*) Omp25 as a critical outer membrane virulence factor that activates the host UPR to promote *Brucella* intracellular survival and proliferation. We demonstrate that Omp25 localizes to the ER and binds the substrate-binding domain (SBD) of BiP, thereby activating all three branches of the UPR, including PERK, IRE1α, and ATF6. This activation also amplifies downstream inflammatory signaling through the BiP–IRE1α–NF-κB axis. *Omp25*-deficient strains exhibit impaired UPR activation and reduced intracellular proliferation and result in an attenuated inflammatory response in mice. These findings reveal a mechanism by which *Brucella* modulates UPR activation and downstream inflammatory response to support chronic infection and highlight Omp25 as a potential target for therapeutic intervention.

## Results

### *B. abortus* Omp25 triggers PERK, IRE1α, and ATF6-mediated UPR signaling

**Figure 1:**
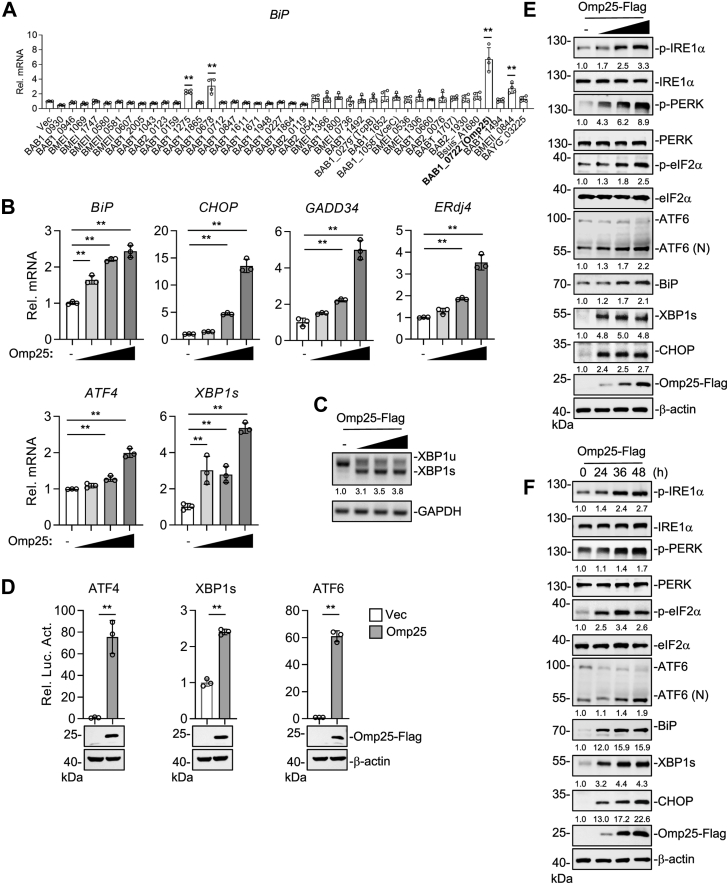
***Brucella abortus* Omp25 activates UPR signaling.***A*, screening of *Brucella* proteins for UPR activation. HEK293T cells (5 × 10^5^) were transfected with empty vector or *Brucella* protein expression plasmids for 24 h before RT–qPCR analysis. *B*, effects of *B. abortus* Omp25 on transcription of UPR downstream genes. HEK293T cells (5 × 10^5^) were transfected with increasing amounts of Omp25-FLAG plasmid (0, 75, 150, or 300 ng) for 24 h before RT–qPCR analysis. *C*, effects of Omp25 on XBP1 mRNA splicing. Total RNA was analyzed by RT–PCR, and XBP1s band intensities were quantified relative to GAPDH. *D*, effects of Omp25 on activation of UPR reporters. HEK293T cells (1 × 10^5^) were cotransfected with Omp25-FLAG (50 ng), pRL-TK (20 ng), and luciferase reporters for ATF6 (20 ng), ATF4 (20 ng), or XBP1s (10 ng). Luciferase activity was measured 24 h post-transfection. *E* and *F*, Omp25 activates PERK, IRE1α, and ATF6 pathways. HEK293T cells (5 × 10^5^) were transfected with increasing doses of Omp25-FLAG (0, 0.3, 0.9, and 2.7 μg) for 24 h (*E*) or with 2 μg Omp25-FLAG for 24, 36, and 48 h (*F*) before Western blotting with the indicated antibodies. Band intensities were quantified relative to β-actin. Data are presented as mean ± SD from n = 3 to 4 independent samples. Statistical significance was determined using one-way ANOVA (*A*, *B*) or unpaired *t* test (*D*). ∗*p* < 0.05, ∗∗*p* < 0.01. ATF, activating transcription factor; HEK293T, human embryonic kidney 293T cell line; IRE1α, inositol-requiring enzyme 1 alpha; Omp, outer membrane protein; PERK, PKR-like ER kinase; qPCR, quantitative PCR; UPR, unfolded protein response; XBP1, X-box binding protein 1; XBP1s, the spliced form of XBP1.

To investigate whether *Brucella* modulates the UPR signaling pathways, we transfected human embryonic kidney 293T (HEK293T) cells with 30 secretory proteins and 11 Omps of *Brucella*, followed by RT–quantitative PCR (qPCR) analysis. Among these candidates, overexpression of Omp25 led to a marked upregulation of *BiP* mRNA expression, which is a marker of UPR activation (Fig. 1*A*). Consistent with this observation, Omp25 also upregulated the expression of several canonical UPR target genes, including *BiP*, *CHOP*, *GADD34*, *ATF4*, *ERdj4*, and *XBP1s*, in a dose-dependent manner (Fig. 1*B*). In addition, the XBP1s was significantly increased (Fig. 1*C*). These results suggest that ectopic expression of Omp25 activates UPR. To exclude nonspecific UPR activation resulting from ER accumulation of bacterial Omps, we analyzed another *Brucella* Omp, BAB2_0076. Although BAB2_0076 showed clear colocalization with the ER, its expression did not induce *BiP* or other UPR marker genes (Fig. S1, *A* and *B*), indicating that ER localization of a bacterial Omp is not sufficient to trigger UPR activation.

The three primary ER stress sensors, PERK, IRE1α, and ATF6, regulate the expression of UPR target genes through their respective downstream transcription factors, ATF4, XBP1s, and ATF6. To determine which of these pathways are activated by Omp25, we performed a dual-luciferase reporter assay in HEK293T cells. Overexpression of Omp25 significantly enhanced the transcriptional activities of ATF4, XBP1s, and ATF6 in reporter assays (Fig. 1*D*). Western blot analysis further demonstrated that Omp25 induced BiP protein expression in both a dose- and time-dependent manner (Fig. 1, *E* and *F*). Moreover, overexpression of Omp25 increased levels of phosphorylated IRE1α (p-IRE1α) and the sXBP1 protein, indicating activation of the IRE1α pathway. It also elevated phosphorylated PERK, phosphorylated eIF2α, and CHOP expression, consistent with PERK pathway activation. In addition, overexpression of Omp25 upregulated the ATF6 (N) level, a marker of ATF6 cleavage and activation (Fig. 1, *E* and *F*). Taken together, these results demonstrate that Omp25 activates UPR signaling through all three major sensors.

### Omp25 induces inflammation *via* the IRE1α–NF-κB signaling axis

**Figure 2:**
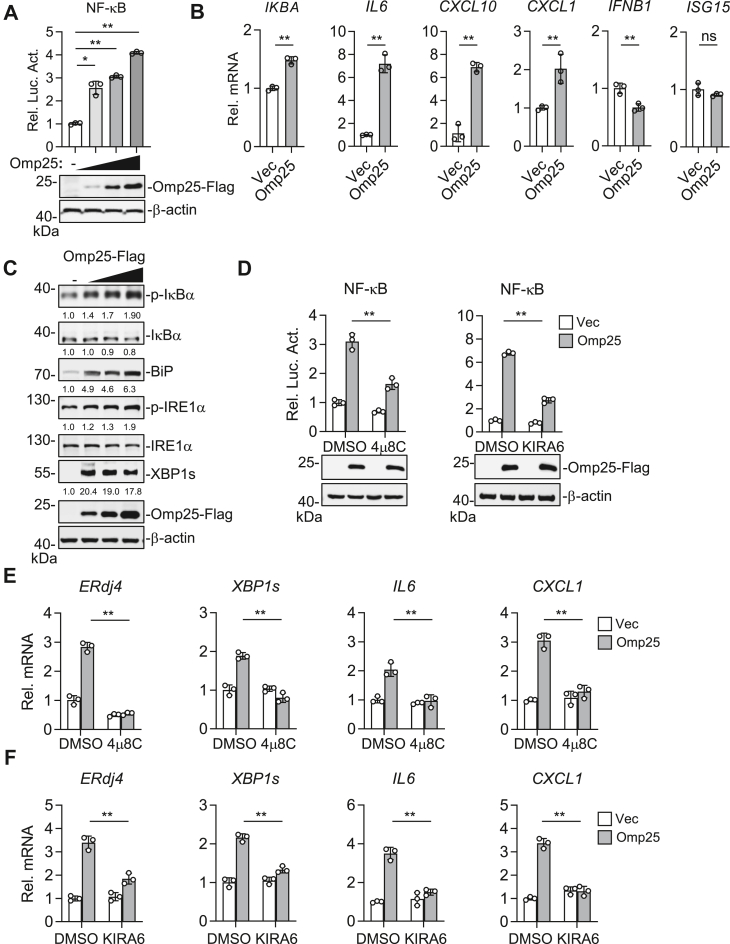
**Omp25 induces the inflammatory response *via* IRE1α activation.***A*, activation of NF-κB by Omp25. HEK293T cells (1 × 10^5^) were cotransfected with NF-κB reporter (20 ng), pRL-TK (20 ng), and increasing amounts of Omp25-FLAG plasmid (25, 50, or 100 ng) for 24 h before luciferase assay. *B*, effects of Omp25 on transcription of inflammatory cytokine genes. HeLa cells (5 × 10^5^) were transfected with empty vector or Omp25-FLAG plasmid for 24 h before RT–qPCR analysis. *C*, activation of the IRE1α–NF-κB signaling pathway by Omp25. HEK293T cells (5 × 10^5^) were transfected with empty vector or Omp25-FLAG plasmid for 24 h before immunoblot analysis. Band intensities were quantified relative to β-actin. *D*, roles of IRE1α RNase and kinase activities in Omp25-induced NF-κB activation. HEK293T cells (1 × 10^5^) were transfected with NF-κB reporter (20 ng), pRL-TK (20 ng), and Omp25-FLAG plasmid (100 ng) for 24 h, followed by treatment with 4μ8C (20 μM, 12 h), KIRA6 (100 nM, 24 h), or DMSO control before luciferase assay. *E* and *F*, effects of 4μ8C (*E*) and KIRA6 (*F*) on Omp25-induced transcription of UPR and inflammatory genes. HeLa cells (5 × 10^5^) were transfected with empty vector or Omp25-FLAG for 24 h. Cells were then treated with 4μ8C (20 μM, 12 h) or KIRA6 (100 nM, 24 h), followed by RT–qPCR analysis. Data are presented as mean ± SD from n = 3 independent samples. Statistical significance was determined using one-way ANOVA (*A*) or two-way ANOVA (*D*–*F*), or unpaired *t* test (*B*). ∗*p* < 0.05, ∗∗*p* < 0.01; ns, not significant. DMSO, dimethyl sulfoxide; HEK293T, human embryonic kidney 293T cell line; IRE1α, inositol-requiring enzyme 1 alpha; KIRA6, kinase-inhibiting RNase attenuator 6; Omp25, outer membrane protein 25; qPCR, quantitative PCR; UPR, unfolded protein response.

Since IRE1α activation can trigger NF-κB signaling, we examined whether Omp25 also promotes NF-κB activation (15). HEK293T cells transfected with Omp25 showed a dose-dependent increase in NF-κB reporter activity (Fig. 2*A*). Consistently, RT–qPCR analysis showed that Omp25 overexpression significantly increased the mRNA levels of inflammatory genes, including *IKBA*, *IL6*, *CXCL10*, and *CXCL1*. In contrast, type I interferon–related genes, such as *IFNB1* and *ISG15*, were not induced by Omp25 (Fig. 2*B*). These results indicate that Omp25 selectively promotes inflammatory responses. Western blot analysis indicated that overexpression of Omp25 increased phosphorylation of IRE1α and IκBα, as well as expression of sXBP1, suggesting simultaneous activation of IRE1α and NF-κB signaling (Fig. 2*C*). The kinase domain of IRE1α is critical for TRAF2 recruitment, whereas its RNase domain is essential for XBP1 mRNA splicing. Both activities can drive NF-κB signaling and inflammatory cytokine expression (15). To confirm that Omp25-induced inflammation depends on IRE1α activity, we treated cells with the IRE1α kinase inhibitor kinase-inhibiting RNase attenuator 6 (KIRA6) or the RNase inhibitor 4μ8C. Omp25-induced NF-κB reporter activity was markedly suppressed by both inhibitors (Fig. 2*D*). Furthermore, RT–qPCR analysis showed that Omp25-induced expression of *ERdj4*, *XBP1s*, *IL6*, and *CXCL1* was reduced upon treatment with either KIRA6 or 4μ8C (Fig. 2, *E* and *F*). Collectively, these results demonstrate that Omp25 promotes inflammatory responses through the IRE1α–NF-κB signaling axis, relying on both the kinase and RNase activities of IRE1α.

### Omp25 engages the SBD of BiP to activate the UPR

**Figure 3:**
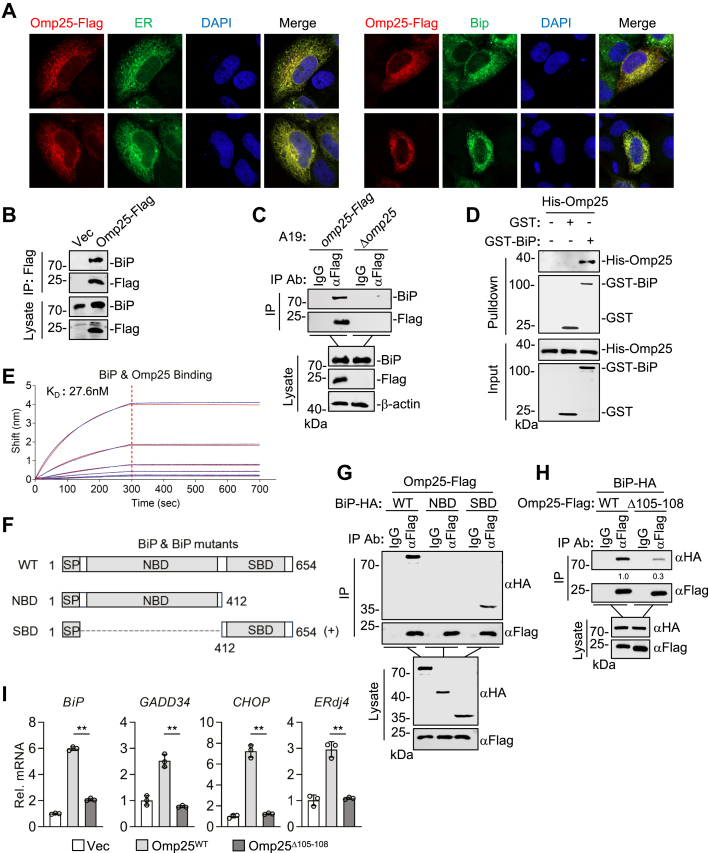
**Omp25 binds to the SBD of Bip to activate UPR.***A*, Omp25 localizes to the ER and colocalizes with BiP. HeLa cells (2 × 10^4^) were cotransfected with Omp25-FLAG and ER marker for 24 h, followed by immunostaining and confocal microscopy. *B*, Omp25 interacts with endogenous BiP. HEK293T cells (2 × 10^7^) were transfected with empty vector or Omp25-FLAG for 24 h. Co-IP was performed using anti-FLAG antibody, followed by immunoblot analysis with the indicated antibodies. *C*, Omp25 interacts with BiP during *Brucella* infection. RAW264.7 cells (5 × 10^7^) infected with A19-*omp25*-FLAG for 48 h were analyzed by co-IP and immunoblot analysis. *D*, direct interaction between Omp25 and BiP. His-Omp25 was incubated with GST-BiP or GST control immobilized on glutathione beads, followed by immunoblot analysis. *E*, binding kinetics of Omp25 and BiP measured by BLI. GST-BiP was immobilized on biosensors, and association and dissociation with Omp25 were monitored in real time. *F*, schematic of BiP full-length and truncated constructs. WT: amino acids 1 to 654; NBD: amino acids 1 to 412; SBD: amino acids 412 to 654. *G*, Omp25 associates with the SBD of BiP. HEK293T cells (1 × 10^7^) transfected with Omp25-FLAG and BiP truncation mutants were analyzed by co-IP and immunoblot analysis with the indicated antibodies. *H*, the interaction of Omp25 mutants with BiP. HEK293T cells (1 × 10^7^) were transfected with WT or mutant Omp25 plasmids. Cells were then subjected to co-IP and immunoblot analysis. Band intensities were quantified by ImageJ. *I*, the effects of Omp25^Δ105–108^ on UPR gene transcription. HEK293T cells (1 × 10^5^) were transfected with empty vector, Omp25^WT^, or Omp25^Δ105–108^ plasmids for 24 h before RT–qPCR analysis. Data are presented as mean ± SD, n = 3 independent samples. Statistical significance was determined by one-way ANOVA; ∗∗*p* < 0.01. BiP, binding immunoglobulin protein; BLI, biolayer interferometry; Co-IP, coimmunoprecipitation; ER, endoplasmic reticulum; GST, glutathione-*S*-transferase; HEK293T, human embryonic kidney 293T cell line; NBD, nucleotide-binding domain; Omp25, outer membrane protein 25; qPCR, quantitative PCR; SBD, substrate-binding domain; UPR, unfolded protein response.

Based on the aforementioned findings, we hypothesized that Omp25 interacts with BiP, the master regulator of all three UPR pathways, and sequesters it away from UPR sensors, thereby triggering UPR activation. Omp25 contains an N-terminal signal sequence, so we first examined its subcellular localization. Confocal microscopy revealed that overexpressed Omp25 colocalized with both an ER marker and endogenous BiP (Fig. 3*A*). Furthermore, coimmunoprecipitation (co-IP) assays demonstrated that overexpressed Omp25 interacted with endogenous BiP (Fig. 3*B*). These results suggest that Omp25 is localized to the ER and associates with BiP.

To determine whether this interaction occurs during *Brucella* infection, we engineered a C-terminal FLAG-tagged Omp25 knock-in strain based on the spontaneously attenuated vaccine strain *B. abortus* A19 (A19-*omp25*-FLAG). Co-IP assays demonstrated that *Brucella*-expressed Omp25 associated with endogenous BiP in cells infected with A19-*omp25*-FLAG (Fig. 3*C*). To confirm a direct interaction between the two proteins, we purified Omp25 and BiP using a prokaryotic expression system and performed glutathione *S*-transferase (GST) pull-down assays. The results indicated that recombinant Omp25 directly bound to GST-tagged BiP but not GST (Fig. 3*D*). Biolayer interferometry (BLI) analysis indicated that Omp25 interacted with BiP with an equilibrium dissociation constant (*K*_*D*_) of 27.6 nM, indicating that they bind to each other with high affinity (Fig. 3*E*).

BiP consists of an N-terminal signal peptide, a nucleotide-binding domain (NBD), and a C-terminal SBD (24). To identify the domain of BiP responsible for interacting with Omp25, we generated BiP truncation mutants containing either the NBD or the SBD (Fig. 3*F*). Co-IP assays revealed that Omp25 specifically interacted with the SBD of BiP (Fig. 3*G*). To further define the Omp25 regions critical for its interaction with BiP, we performed molecular docking analysis using AlphaFold, which predicted that residues S105 and G108 in Omp25 are critical for binding the SBD domain of BiP (Fig. S2). Based on this prediction, we constructed a deletion mutant lacking amino acids 105 to 108 of Omp25 (Omp25^Δ105–108^). Co-IP assays revealed that Omp25^Δ105–108^ had markedly reduced ability for binding to BiP compared with WT Omp25 (Fig. 3*H*). Consistently, RT–qPCR showed that Omp25^Δ105–108^ had a decreased ability to induce UPR target genes compared with WT Omp25 (Fig. 3*I*). These findings suggest that Omp25 activates UPR signaling by directly interacting with BiP through their specific motifs.

### Omp25 disrupts BiP interactions with PERK, IRE1α, and ATF6

**Figure 4:**
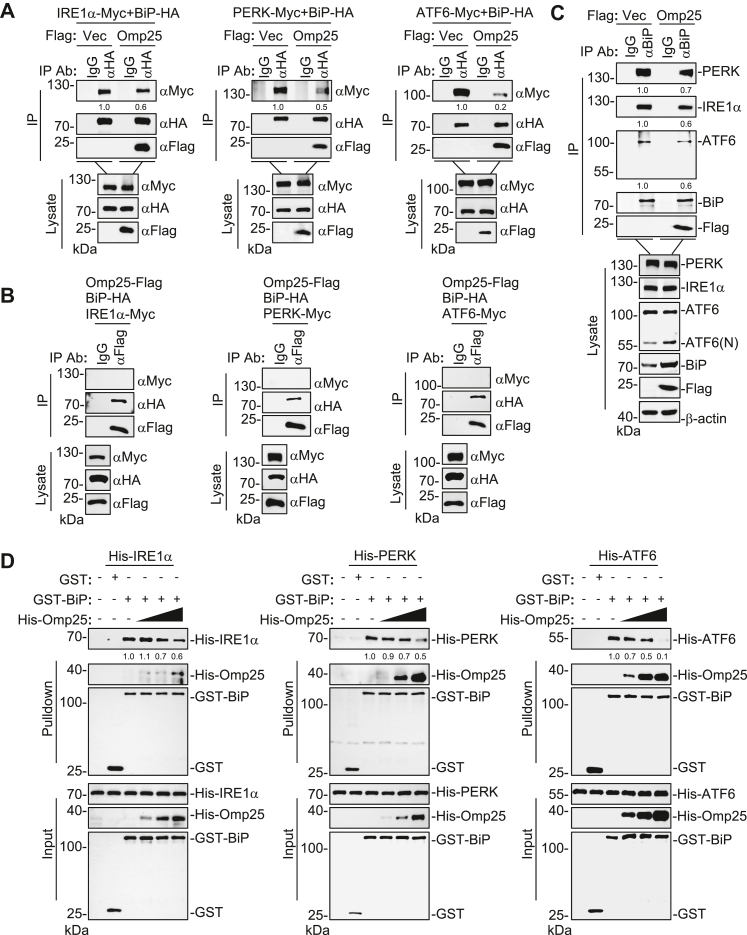
**Omp25 disrupts BiP interactions with PERK, IRE1α, and ATF6.***A*, effects of Omp25 on the association of BiP with PERK, IRE1α, and ATF6. HEK293T cells (1 × 10^7^) were transfected with the indicated plasmids for 24 h, followed by co-IP and immunoblot analysis. *B*, Omp25 interacts with BiP but not with PERK, IRE1α, or ATF6. HEK293T cells (1 × 10^7^) were transfected with the indicated plasmids for 24 h, followed by co-IP and immunoblot analysis. *C*, effects of Omp25 on the endogenous association of BiP with PERK, IRE1α, and ATF6. HEK293T cells (1 × 10^7^) transfected with empty vector or Omp25-FLAG were analyzed by co-IP using anti-BiP antibody, followed by immunoblot analysis. *D*, effects of Omp25 on the association of BiP with PERK, IRE1α, and ATF6 in GST pull-down assays. Increasing amounts of recombinant His-Omp25 were incubated with GST-BiP and His-tagged luminal domains of PERK, ATF6, or IRE1α, followed by GST pull-down and immunoblot analysis. Band intensities were quantified by ImageJ. ATF6, activating transcription factor 6; BiP, binding immunoglobulin protein; Co-IP, coimmunoprecipitation; GST, glutathione *S*-transferase; HEK293T, human embryonic kidney 293T cell line; IRE1α, inositol-requiring enzyme 1 alpha; Omp25, outer membrane protein 25; PERK, PKR-like ER kinase.

Given our findings that Omp25 binds the SBD of BiP, we investigated whether this interaction facilitates the release of the ER stress sensors PERK, IRE1α, and ATF6. Co-IP assays demonstrated that Omp25 markedly reduced the association between BiP and each of these sensors (Fig. 4*A*). In parallel, no detectable association between Omp25 and PERK, IRE1α, or ATF6 was observed in co-IP assays (Fig. 4*B*), suggesting that Omp25 does not form a stable ternary complex with these UPR sensors. Consistent with this, ectopic expression of Omp25 reduced the endogenous association of BiP with PERK, IRE1α, and ATF6 (Fig. 4*C*). These results suggest that Omp25 promotes the dissociation of BiP from the ER stress sensors.

Since PERK, IRE1α, and ATF6 are ER transmembrane proteins whose luminal domains (LDs) are responsible for BiP binding, we further explored this mechanism using an *in vitro* system. We purified the LDs of PERK (PERK^LD^), IRE1α (IRE1α^LD^), and ATF6 (ATF6^LD^), along with BiP produced in a prokaryotic system. GST pull-down assays indicated that Omp25 decreased the binding of BiP to PERK^LD^, IRE1α^LD^, and ATF6^LD^ in a dose-dependent manner (Fig. 4*D*). Collectively, these findings demonstrate that Omp25 engages BiP and disrupts its interactions with PERK, IRE1α, and ATF6, thereby promoting the release and activation of these ER stress sensors to initiate UPR signaling.

### Omp25 deficiency attenuates *Brucella*-induced UPR and inflammatory responses

**Figure 5:**
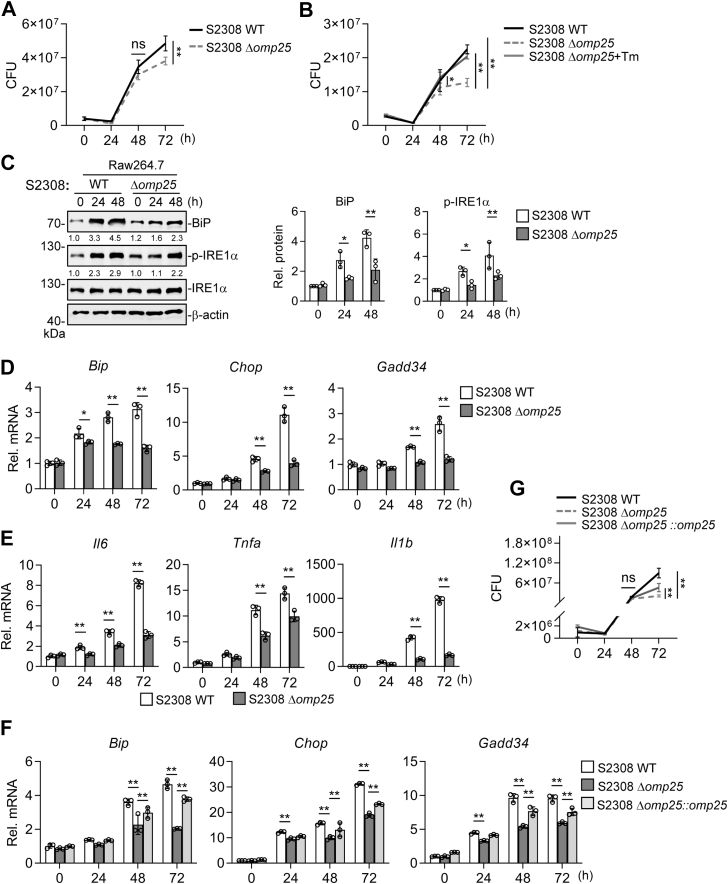
**Omp25 deficiency attenuates *Brucella abortus* S2308-induced UPR and inflammatory responses.***A*, intracellular replication of *B. abortus* S2308 WT and Δ*omp25* strains. RAW264.7 cells (5 × 10^5^) were infected at MOI = 200 for 1 h, washed, and incubated in Dulbecco's modified Eagle's medium with 1% fetal bovine serum. The number of viable intracellular bacteria was determined at the indicated time points. *B*, Tunicamycin (Tm) pretreatment partially restores Δ*omp25* intracellular replication. Cells were pretreated with 0.01 μg/ml Tm for 30 min before infection (MOI = 200), then processed as in (*A*) for CFU enumeration. *C*, effects of *omp25* deletion on *Brucella*-induced UPR. RAW264.7 cells (1 × 10^6^) were left uninfected or infected (MOI = 200) with the WT or Δ*omp25* strain. Lysates were analyzed by immunoblotting with the indicated antibodies. Band intensities were quantified by ImageJ. *D* and *E*, effects of *omp25* deletion on UPR signaling and inflammatory cytokine gene expression. RAW264.7 cells (5 × 10^5^) were infected (MOI = 1000) with WT or Δ*omp25* strain for the indicated times, followed by RT–qPCR analysis. *F*, reconstitution of UPR signaling by *omp25* complementation. RAW264.7 cells (5 × 10^5^) were infected (MOI = 1000) with WT, Δ*omp25*, or Δ*omp25*::*omp25* strain for the indicated times, followed by RT–qPCR analysis. *G*, effects of *omp25* complementation on the bacterial burden. RAW264.7 cells (5 × 10^5^) were infected (MOI = 200) with the indicated strains, and the number of viable intracellular bacteria was determined. Data shown in *A*–*G* are presented as mean ± SD from three independent experiments. Statistical significance was determined by two-way ANOVA; ∗*p* < 0.05, ∗∗*p* < 0.01; ns, not significant. MOI, multiplicity of infection; Omp25, outer membrane protein 25; qPCR, quantitative PCR; UPR, unfolded protein response.

To investigate the roles of Omp25-mediated UPR activation upon *Brucella* infection, we generated *omp25*-deleted strains (Δ*omp25*) and the corresponding *omp25*-complemented strain (Δ*omp25*::*omp25*) in both the attenuated *B. abortus* A19 strain and the virulent S2308 strain (Fig. S3*A*). Western blot analysis confirmed the absence of Omp25 in both bacterial cultures and infected-cell lysates from the *omp25* deletion mutants, whereas Omp25 expression was restored in the complemented strain (Fig. S3, *B*–*D*). We next examined the growth kinetics of the WT and the Δ*omp25* strains. No significant differences were observed in their growth curves in culture medium, indicating that Omp25 is not required for extracellular replication (Fig. S3*E*). During intracellular infection, the Δ*omp25* strain exhibited similar proliferation rates to their respective WT strain at 24 and 48 h postinfection. However, a significant reduction in colony-forming units (CFUs) was observed at 72 h for both Δ*omp25* strains (Figs. 5*A* and S4*A*). These results suggest that Omp25 contributes to sustained intracellular survival and proliferation.

To assess whether the intracellular replication defect of the Δ*omp25* strains is associated with impaired UPR activation, infected cells were pretreated with tunicamycin (Tm), a well-established UPR activator. Tm treatment partially restored the intracellular replication of the Δ*omp25* strains (Figs. 5*B* and S4*B*), supporting a functional link between UPR activation and *Brucella* intracellular survival.

Consistent with this observation, analysis of UPR markers revealed that BiP and p-IRE1α levels were markedly reduced in cells infected with the Δ*omp25* strains compared with the corresponding WT strains (Figs. 5*C* and S4*C*). RT–qPCR further showed that transcriptional induction of *Bip*, *Chop*, and *Gadd34* was significantly attenuated in cells infected with the Δ*omp25* strains at 48 and 72 h postinfection compared with their respective WT strains (Figs. 5*D* and S4*D*). Moreover, cells infected with the Δ*omp25* strains exhibited lower induction of inflammatory genes, such as *Il6*, *Tnfa*, and *Il1b*, compared with their respective WT strains (Figs. 5*E* and S4*E*).

Finally, complementation of *omp25* partially restored UPR activation and intracellular replication in the S2308 Δ*omp25* strain (Fig. 5, *F* and *G*). Collectively, these findings suggest that Omp25 is important for intracellular proliferation of *B. abortus* as well as the activation of the UPR and inflammatory responses during infection.

### Omp25 deletion reduces *Brucella*-induced UPR and inflammation *in vivo*

**Figure 6:**
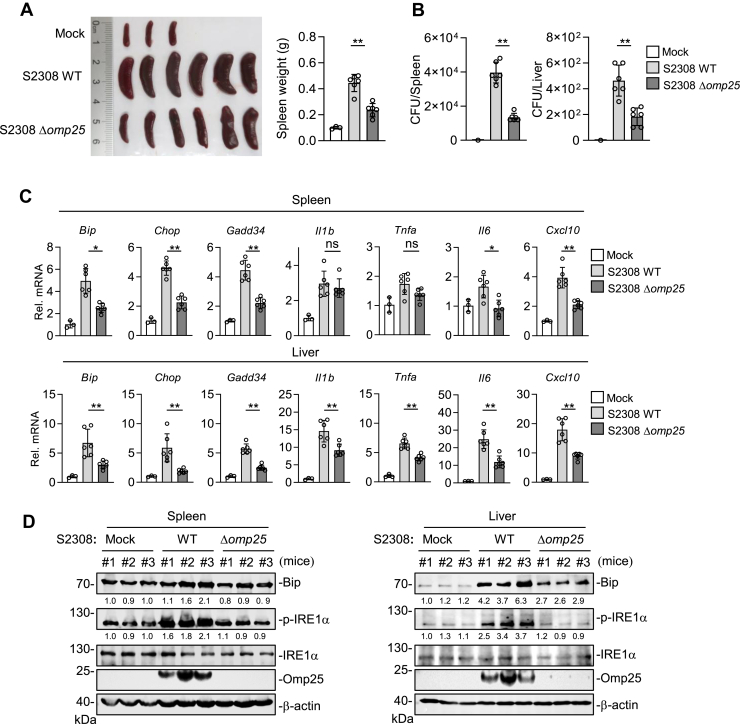
***Omp25* deletion reduces *Brucella*-induced UPR and inflammation *in vivo.****A*, effects of *omp25* deficiency on spleen morphology. Six- to 8-week-old BALB/c mice were intraperitoneally infected with *Brucella abortus* S2308 WT or Δ*omp25* strain (3 × 10^5^ CFU). Spleens were harvested 20 days postinfection for gross morphology and weight measurements. *B*, bacterial burdens in spleen and liver tissues. Spleen and liver samples were harvested at 20 days postinfection, and the number of viable intracellular bacteria was determined. *C*, effects of *omp25* deficiency on UPR and inflammatory gene expression in infected tissues. Spleen and liver samples were collected at 20 days postinfection and analyzed by RT–qPCR for the indicated genes. *D*, effects of *omp25* deficiency on UPR signaling pathway activation in infected mouse tissues. Spleen and liver lysates were subjected to immunoblot analysis using the indicated antibodies. Band intensities were quantified by ImageJ and normalized to β-actin. Data in *A*–*C* are presented as mean ± SD. Mock, n = 3; WT and Δ*omp25*, n = 6 mice per group. Statistical analysis was performed using one-way ANOVA; ∗*p* < 0.05, ∗∗*p* < 0.01; ns, not significant. CFU, colony-forming unit; Omp25, outer membrane protein 25; qPCR, quantitative PCR; UPR, unfolded protein response.

To investigate the role of Omp25 in *B. abortus* virulence and *in vivo* proliferation, we infected mice with the WT or Δ*omp25* strain. Mice infected with S2308 WT exhibited marked splenomegaly and increased spleen weights, whereas these effects were significantly attenuated in mice infected with the Δ*omp25* strain (Fig. 6*A*). We then assessed bacterial burdens and histopathological changes in the spleen and liver. Compared with WT-infected mice, bacterial loads in both organs were significantly reduced in the Δ*omp25*-infected group (Fig. 6*B*). Histopathological analysis further revealed that splenic swelling, serous exudation, and red blood cell dispersion were notably alleviated in Δ*omp25*-infected mice (Fig. S5), indicating reduced tissue damage and inflammation in Δ*omp25* strain compared with WT-infected mice. These results are consistent with a previous report showing that *omp25* deficiency attenuates *Brucella* virulence and reduces its *in vivo* proliferation (22).

To determine whether *omp25* deficiency impairs *B. abortus*–induced UPR and inflammatory responses *in vivo*, we analyzed gene expression in the spleen and liver of infected mice. Although UPR-related genes, such as *Bip*, *Chop*, and *Gadd34*, were upregulated in the spleen and liver of both WT and Δ*omp25-*infected mice, their expression levels were significantly lower in mice infected with Δ*omp25* compared with the WT strain (Fig. 6*C*). Similarly, transcripts of proinflammatory cytokines, such as *Il1b*, *Tnfa*, *Il6*, and *Cxcl10*, were markedly reduced in Δ*omp25*-infected mice (Fig. 6*C*). Western blot analysis also demonstrated that levels of BiP and p-IRE1α were substantially decreased in the spleen and liver of Δ*omp25*-infected mice compared with those infected with S2308 WT (Fig. 6*D*). Collectively, these findings suggest that Omp25 enhances *Brucella* proliferation and pathogenesis by promoting UPR activation and downstream inflammatory responses *in vivo*.

### Omp25 promotes severe placentitis by enhancing UPR activation during *Brucella* infection

**Figure 7:**
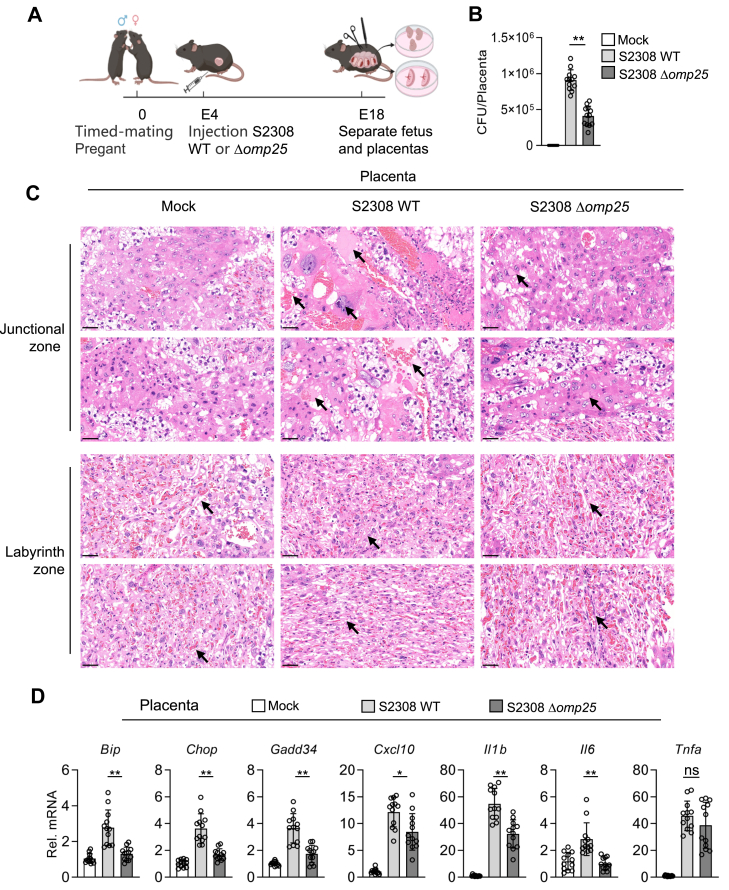
**Omp25 promotes severe placentitis by enhancing UPR activation during *Brucella* infection.***A*, schematic diagram illustrating the placental infection model in pregnant mice (https://www.biorender.com). *B*, effects of *omp25* deficiency on bacterial load in placentas. Placentas were harvested from C57BL/6 pregnant mice at 14 days postinfection with *Brucella abortus* S2308 WT or Δ*omp25* strain (1.5 × 10^5^ CFU), and the number of viable intracellular bacteria was determined. *C*, histopathological analysis of placental tissues. Placentas were harvested at 14 days postinfection and subjected to H&E staining to assess inflammation and tissue damage. The representative micrographs for the indicated group are derived from two microscopic fields of the same tissue sample. The scale bars represent 50 μm. *D*, effects of *omp25* deficiency on transcription of UPR and inflammatory genes in placentas. Total RNA was extracted from placental tissues at 14 days postinfection and analyzed by RT–qPCR for the indicated genes. Data shown in (*B*) and (*D*) are presented as mean ± SD, n = 12 independent samples. Statistical analysis was performed using one-way ANOVA; ∗*p* < 0.05, ∗∗*p* < 0.01; ns, not significant. CFU, colony-forming unit; Omp25, outer membrane protein 25; qPCR, quantitative PCR; UPR, unfolded protein response.

*Brucella* typically elicits mild chronic inflammation in experimentally infected mice or its natural hosts (25, 26). In contrast, in pregnant ruminants, such as sheep and goats, it induces severe acute placentitis leading to abortion (27). To investigate the role of Omp25 in this pathological process, we employed a pregnant mouse model. As illustrated in Figure 7*A*, 4-day pregnant mice were infected with either the *B. abortus* S2308 WT or Δ*omp25* strain, and placentas along with fetuses were collected at 14 days postinfection. Quantification of bacterial burdens revealed that placental bacterial loads were significantly lower in mice infected with the S2308 Δ*omp25* strain compared with those infected with WT strain (Fig. 7*B*). Histopathological analysis of the placental junctional and labyrinth zones further highlighted the effects of *omp25* deletion. In the junctional zone, placentas from WT-infected mice exhibited pronounced pathological changes, including dense serous exudation and areas of necrosis. In contrast, these pathological features were substantially alleviated in placentas from Δ*omp25*-infected mice. Examination of the labyrinth zone revealed that trophoblast cells in Δ*omp25*-infected placentas were more loosely organized with enlarged vascular spaces, indicative of improved maternal–fetal blood flow and a more favorable environment for nutrient exchange relative to WT-infected placentas (Fig. 7*C*).

To determine whether the reductions in the bacterial burden and placental pathology of S2308 Δ*omp25*–infected mice were associated with attenuated UPR and inflammatory responses, we assessed the transcriptional profiles of UPR- and inflammation-related genes in placental tissues. Expression levels of *Bip*, *Chop*, and *Gadd34*, as well as proinflammatory genes, were markedly lower in placentas from S2308 Δ*omp25*–infected mice than those infected with WT (Fig. 7*D*). Collectively, these findings demonstrate that Omp25 promotes *Brucella* proliferation as well as UPR activation and inflammatory responses in the placenta, thereby exacerbating placentitis and increasing the risk of abortion.

## Discussion

*Brucellosis* is one of the most widespread and clinically important zoonotic diseases, especially in developing countries (28). During infection, *Brucella* forms rBCVs that fuse with the ER, establishing a niche that supports bacterial replication (29). This process involves extensive remodeling of ER architecture and disruption of ER homeostasis, ultimately activating the UPR. Although bacterial Omps are known to mediate host–pathogen interactions, their roles in modulating ER function remain poorly understood. Elucidating how Omps interact with the ER is crucial for uncovering the mechanisms underlying the chronic infection and immune evasion of *Brucella*.

A dynamic, bidirectional crosstalk between pathogen and host drives both disease initiation and progression. In this study, we demonstrate that *B. abortus* Omp25 activates the UPR by inducing BiP and multiple UPR target gene expression, thereby modulating ER homeostasis. As a conserved structural component essential for *B. abortus* virulence and intracellular survival, Omp25 was found to activate all three major branches of the UPR pathway. Upon activation, IRE1α oligomerizes, activating its cytoplasmic kinase and RNase domains. This leads to the splicing of XBP1 mRNA and subsequent production of the transcription factor XBP1s, which drives the expression of inflammatory cytokines. In addition, IRE1α can recruit TRAF2 to form a signaling complex that activates NF-κB, further amplifying the inflammatory response (30, 31). Furthermore, our data show that inhibiting either the kinase or RNase activity of IRE1α suppresses Omp25-induced NF-κB activation, indicating that Omp25-mediated inflammation depends on activated IRE1α. To further evaluate the role of IRE1α in Omp25-induced NF-κB activation, we used the kinase inhibitor KIRA6. Although higher concentrations of KIRA6 may affect other kinases, such as p38 mitogen-activated protein kinase (MAPK), studies indicate that at 100 nM, KIRA6 selectively attenuates IRE1α activity without broadly inhibiting MAPK signaling (32). At this concentration, KIRA6 effectively abolished the enhanced NF-κB activity driven by Omp25, suggesting that the observed effects primarily reflect inhibition of IRE1α rather than off-target kinase activity. These findings, together with complementary genetic and pharmacological approaches, support a central role for IRE1α-dependent signaling in mediating Omp25-induced inflammatory responses.

Since Omp25 activates all three UPR sensors, we hypothesized that it functions upstream by modulating BiP interactions. Confocal microscopy confirmed that Omp25 localizes to the ER and colocalizes with BiP. GST pull-down and BLI assays using purified proteins demonstrated a direct interaction between Omp25 and BiP. Furthermore, domain mapping revealed that Omp25 binds specifically to the SBD of BiP. Under homeostatic conditions, BiP associates with the LDs of IRE1α and PERK *via* its NBD and with ATF6 *via* its SBD. Upon accumulation of misfolded proteins, binding to BiP’s SBD induces an allosteric change that disrupts BiP’s interactions with all three UPR sensors (24). Omp25 adopts a β-barrel structure, and molecular docking analysis indicates that it interacts with SBD through residues S105 and G108. Deletion of amino acids 105 to 108 impaired the ability of Omp25 to bind BiP and activate the UPR. Consistently, both overexpression studies and GST pull-down assays confirmed that Omp25 promotes BiP dissociation from PERK, IRE1α, and ATF6. These results suggest that this dissociation is not a consequence of UPR activation but rather a direct result of the sequestration of BiP from the UPR sensors by Omp25.

We also examined the functional significance of Omp25 during infection *in vitro*. *B. abortus* Δ*omp25* strain exhibited normal growth in liquid culture, consistent with previous findings in *Brucella melitensis* Δ*omp25* (33). Although bacterial loads of the mutant strains were comparable to the WT strain before 48 h postinfection, a significant reduction was observed by 72 h. These results suggest that Omp25 is not essential for initial infection or replication but plays a critical role in sustaining intracellular survival or proliferation. Furthermore, *B. abortus* WT strain induced significantly higher expression of UPR-related genes and proinflammatory cytokines compared with the Δ*omp25* strain, indicating that Omp25-mediated UPR activation contributes to *Brucella*’s multiplication within host cells. Supporting this interpretation, pharmacological induction of the UPR with Tm partially restored the intracellular burden of the Δ*omp25* strain to levels approaching those of the WT strain. This rescue occurred despite the absence of Omp25, indicating that the reduced late-stage replication of Δ*omp25* reflects insufficient UPR activation rather than intrinsic bacterial growth defects. Similarly, genetic complementation of *omp25* increased UPR activation and partially rescued intracellular bacterial loads. These functional rescue experiments provide direct evidence that Omp25 sustains intracellular replication through active modulation of host UPR signaling.

Finally, we investigated the effects of *omp25* deficiency using a mouse model. Compared with the *B. abortus* WT strain, mice infected with the Δ*omp25* strain exhibited significantly reduced bacterial loads in multiple tissues, along with attenuated induction of UPR signaling and inflammatory cytokine expression. The pathogenicity of *Brucella* largely depends on its ability to persist and replicate within host phagocytes (*e.g.*, macrophages, dendritic cells) and placental trophoblasts (34, 35). In pregnant animals, placental macrophages produce excessive inflammatory cytokines, contributing to placentitis and abortion (36, 37). In our pregnant mouse model, the Δ*omp25* strain induced lower transcriptional levels of UPR-related and proinflammatory genes in placental tissues, further supporting that Omp25 plays a role in mediating inflammation and persistence. This is consistent with a previous study showing that *Brucella* Omp25 is involved in the activation of the MAPK pathway and induction of inflammatory cytokines (38). Previous studies have reported that Δ*omp25* strains are attenuated *in vivo*, primarily because of impaired intracellular survival (22). Our data extend these findings by showing that defective UPR activation occurs early during infection, before measurable reductions in the bacterial burden. This temporal separation suggests that impaired host UPR modulation is an upstream event contributing to reduced persistence, rather than a secondary consequence of lower bacterial numbers. While type IV secretion system effectors, such as VceC, can activate IRE1α-dependent UPR during placental infection to support *Brucella* replication (39), Omp25 represents an additional mechanism. By directly disrupting BiP–sensor interactions, Omp25 creates a permissive ER environment that facilitates sustained intracellular survival and may enhance downstream effector-mediated signaling, highlighting a layered and temporally coordinated strategy of UPR modulation by *Brucella*.

Previous studies have primarily characterized Omp25 as an immunomodulatory virulence factor that suppresses Toll-like receptor– and cGAS-STING–mediated signaling to dampen early host inflammatory responses and facilitate immune evasion (23, 40, 41). Conversely, other reports have noted that Omp25 can promote proinflammatory cytokine production and activate MAPK pathways in specific cellular contexts, although the underlying mechanisms remain unclear (38, 42). Our findings reconcile these seemingly contradictory observations by identifying UPR activation as a previously unrecognized mechanism of Omp25-mediated host modulation. In contrast to classical pattern recognition receptor pathways that dominate early infection, UPR signaling represents an intracellular stress-sensing system that becomes engaged during sustained bacterial replication. Through direct interaction with the ER chaperone BiP, Omp25 activates the IRE1α pathway, leading to NF-κB activation and inflammatory amplification. We therefore propose that Omp25 acts as a stage- and pathway-specific immunomodulator, suppressing innate immune signaling to evade early host defenses while engaging UPR signaling to enhance intracellular adaptation, inflammation, and persistence during later stages of infection.

In conclusion, this study identifies *B. abortus* Omp25 as a critical virulence factor that activates the UPR by directly binding BiP and promoting its dissociation from PERK, IRE1α, and ATF6. This interaction initiates downstream UPR signaling, thereby supporting the intracellular proliferation of *Brucella*. Overall, these results offer new mechanistic insights into the roles of bacterial Omps in UPR regulation and underscore Omp25 as a potential target for therapeutic intervention and vaccine development against chronic *Brucella* infection.

## Experimental procedures

### Ethics statement

All experiments with live *B. abortus* S2308 (CVCC788), A19 (CVCC70202), or their derivatives were conducted at the Biosafety Level 3 laboratory of the Lanzhou Veterinary Research Institute (LVRI), Chinese Academy of Agricultural Sciences. This laboratory is accredited by the China National Accreditation Service for Conformity Assessment and the Gansu Provincial Department of Agriculture and Rural Affairs. All animal experiments were approved by the Committee for the Ethics of Animal Experiments of the Lanzhou Veterinary Research Institute at the Chinese Academy of Agricultural Sciences (approval number: LVRIAEC-2025-015).

### Cells, bacterial strains, reagents, and antibody

HEK293T, HeLa, and RAW264.7 cells were obtained from the American Type Culture Collection. RAW264.7 mouse macrophages were cultured in high-glucose Dulbecco's modified Eagle's medium (C11995500BT, Invitrogen) supplemented with 10% fetal bovine serum (FBS-E020124, NEWZERUM). HEK293T cells and HeLa cells were cultured in high-glucose Dulbecco's modified Eagle's medium (C11965500BT, Gibco) with 10% fetal bovine serum (SA211.02, CellMax) and streptomycin (SV30010, HyClone).

The *B. abortus* S2308 and A19 were from LVRI. The *Escherichia coli* DH5α and *E. coli* BL21 (DE3) used in the experiments were prepared by the laboratory for cloning and protein expression, respectively.

The Protein A+G Agarose (P2055, Beyotime), PMSF (P7626, Sigma), Dual-Specific Luciferase Assay Kit (E1980, Promega); SYBR Green supermix (Q312, Vazyme); HiScript II Select RT SuperMix for qPCR (R323, Vazyme); the ClonExpress Ultra One Step Cloning Kit for PCR (C115, Vazyme); the inhibitors aprotin, leupeptin, β-glycerophosphate disodium salt, and sodium orthovanadate (HY-P0017, HY-18234A, HY-126304, and HY-D0852, MCE); 4′,6-diamidino-2-phenylindole (C1002, Beyotime); His-tag protein purification kit (P2226, Beyotime); glutathione resin (L00206, GenScript); bovine serum albumin (A8020, Solarbio); RNAiso Plus reagent (9109, Takara); Tm (12819S, Cell Signaling Technology), and FuGENEHD (HD-1000, Promega) were purchased from the indicated manufacturers.

Mouse monoclonal antibodies against HA (66006, Proteintech); rabbit monoclonal antibodies against HA (H6908, Sigma); mouse monoclonal antibodies against FLAG (F3165, Sigma); horseradish peroxidase (HRP)-FLAG (ZB15939, Servicebio); rabbit polyclonal antibodies against Myc (16286-1-AP, Proteintech); HRP-His (HRP-66005, Proteintech); IgG (I5381 and I5006, Sigma); BiP (11587-1-AP, Proteintech); phosphorylated PERK (29546-1-AP, Proteintech); p-IRE1α (Ab124945, Abcam); IRE1α (3294S, Cell Signaling Technology); PERK (20582-1-AP, Proteintech); phosphorylated eIF2α (3398S, Cell Signaling Technology); eIF2α (11170-1-AP, Proteintech); ATF6 (ab122897, Abcam); XBP1s (24868-1-AP, Proteintech); CHOP (15204-1-AP, Proteintech); p-IκBα (9246S, Cell Signaling Technology); IκBα (9242S, Cell Signaling Technology); GST (66001-2-Ig, Proteintech); β-actin (A2228, Sigma–Aldrich); anti-mouse IgG (H+L), F(ab')2 fragment (Alexa Fluor 594 Conjugate) (8890, Cell Signaling Technology); Alexa Fluor 555 goat anti-rabbit IgG (H+L) (A-21428, Invitrogen), and Alexa Fluor 488 goat anti-rabbit IgG (H+L) (A11008, Invitrogen) were purchased from the indicated manufacturers.

### Plasmids

*Brucella* protein expression constructs, including *omp25*-FLAG and its mutants, BiP-HA and its mutants, as well as Myc-tagged IRE1α, PERK, and ATF6, were generated using standard molecular biology techniques. The XBP1s luciferase reporter plasmid was purchased from Beyotime.

### Transfection and reporter assays

HEK293T cells were transfected with the indicated plasmids using the calcium phosphate precipitation method. To control for transfection efficiency, reporter plasmids (Firefly luciferase) were cotransfected with pRL-TK (Renilla luciferase) plasmid. Twenty-four hours post-transfection, cells were either left untreated or subjected to the indicated stimuli. Luciferase activity was measured using a dual-luciferase reporter assay system according to the manufacturer’s protocol. Firefly luciferase activity was normalized to Renilla luciferase activity to account for transfection efficiency.

### Generation of *Brucella* gene deletion, FLAG-tagged knock-in, and complementation recombinant strains

*B. abortus* S2308 and A19 Δ*omp25* strains were constructed by double homologous recombination. Briefly, ∼500 bp regions upstream (left homology arm, LA) and downstream (right homology arm, RA) of the *omp25* gene were amplified and cloned into the kanamycin-resistant vector pUC19 containing the *sacB* gene as a counter-selectable marker (pUC19-*sacB*-LA-RA), using the ClonExpress Ultra One Step Cloning Kit. The constructs were verified by restriction enzyme digestion and Sanger sequencing.

Recombinant plasmids were electroporated into competent *B. abortus* cells. Following 24 h of recovery in antibiotic-free tryptic soy broth, transformants were selected on tryptic soy agar (TSA) plates containing kanamycin to identify colonies harboring single-crossover (first homologous recombination) events. For the selection of double-crossover (second recombination) mutants, colonies were plated on TSA supplemented with 15% sucrose. Due to the presence of the *sacB* gene, which encodes the sucrose-sensitive enzyme levansucrase, only plasmid-excised recombinants could survive under these conditions. Deletion of *omp25* was confirmed by PCR and Western blot analysis.

The A19-*omp25*-FLAG strain was generated by replacing the endogenous *omp25* with an epitope-tagged version *via* a similar strategy. A recombinant plasmid (pUC19-*sacB*-*omp25*-FLAG-RA) was constructed and introduced into A19 cells, followed by the same selection procedures. Successful integration was validated by PCR and Western blotting.

For the S2308 Δ*omp25*::*omp25* strain, the Omp25 gene was cloned into the shuttle vector pBBR1MCS-2 to generate pBBR1MCS-2-*omp25*-FLAG (43, 44). The plasmid was electroporated into competent cells of the *omp25*-deletion strain. After 24 h of recovery in antibiotic-free tryptic soy broth, cultures were plated on kanamycin-containing TSA to select complemented strains. Expression of Omp25-FLAG was confirmed by Western blotting. The sequences of the PCR primers are shown in Table S1.

### Protein expression, purification, and antibody preparation

A DNA fragment coding for *B. abortus* Omp31b (amino acids 28–261), the ER-LD of UPR sensors-PERK^LD^ (amino acids 55–514), or IRE1α^LD^ (amino acids 19–443) was constructed into the pET-30c vector. A DNA fragment coding for ATF6^LD^ (amino acids 399–670) or Omp25 was constructed into the pET-28a-sumo vector. A DNA fragment coding for BiP (amino acids 20–654) was constructed into the pGEX-6p-1 vector. The primer sequences are shown in Table S1.

The plasmids were transformed into *E. coli* BL21–competent cells for protein expression. Cells were harvested by centrifugation, resuspended in cold lysis buffer, and lysed by sonication. After centrifugation, the supernatant was subjected to nickel-affinity chromatography using a His-tag protein purification kit for His-tagged proteins. BiP was purified using GST beads and eluted with reduced glutathione. Recombinant proteins were quantified using bovine serum albumin.

The recombinant Omp25 and Omp31b proteins were used as antigens to immunize mice, and polyclonal antisera were collected. The resulting anti-Omp25 and anti-Omp31b sera specifically recognized their respective target proteins.

### GST pull-down assay

To assess the interaction between BiP and Omp25, purified His-tagged Omp25 (His-Omp25) protein was incubated with either GST alone or GST-tagged BiP immobilized on glutathione-Sepharose beads at 4 °C for 3 h. After incubation, the beads were washed three times with GST pull-down buffer (50 mM Tris–HCl, 400 mM NaCl, 10 mM MgCl_2_, 1% NP-40, 1 mM EDTA, 1 mM DTT, pH 8.0). Following centrifugation, the supernatant was discarded, and the bead pellets were resuspended in SDS sample buffer before being subjected to Western blot analysis.

To determine whether Omp25 influences the interaction between BiP and the ER stress sensors, PERK, IRE1α, or ATF6, purified GST-BiP was incubated at 4 °C for 2 h with purified LDs of PERK (His-PERK^LD^), IRE1α (His-IRE1α^LD^), or ATF6 (His-ATF6^LD^), respectively, and the beads were washed three times with GST pull-down buffer. Increasing amounts of His-Omp25 protein were added to the reaction mixtures to assess competition. Subsequent steps, including incubation, washing, and immunoblotting, were performed as described above (45).

### RT–qPCR

RT–qPCR was performed as previously described (46). Briefly, total RNA was extracted from cells using RNAiso Plus reagent according to the manufacturer’s instructions. RT was carried out to synthesize complementary DNA using a standard RT kit. Quantitative PCR was then performed using gene-specific primers to assess the mRNA levels of target genes. Relative expression levels were calculated using the ΔΔCt method and normalized to human *GAPDH* or mouse *Gapdh* as internal controls. The sequences of all primers used are listed in Table S1.

### Co-IP and immunoblot analysis

Cells were lysed on ice with lysis buffer (50 mM Tris–HCl, pH 7.4; 1% NP-40; 150 mM NaCl; and complete protease inhibitor cocktail) at 4 °C for 10 min, followed by brief sonication. Lysates were cleared by centrifugation at 13,000 rpm for 10 min at 4 °C. The resulting supernatants were incubated with the indicated antibodies and Protein A+G agarose beads at 4 °C for 3 h.

Beads were washed four times with a high-stringency washing buffer (50 mM Tris–HCl, pH 7.4; 1% NP-40; and 500 mM NaCl). Immunoprecipitated proteins were eluted in SDS sample buffer, resolved by SDS-PAGE, and subjected to immunoblotting with the indicated primary antibodies.

### Immunofluorescence assay

HeLa cells were seeded on coverslips in 24-well plates and transfected with the indicated plasmids using FuGENE HD for 24 h. Cells were then fixed with 4% paraformaldehyde for 30 min at room temperature and washed three times with PBS. Following fixation, cells were permeabilized with 0.25% Triton X-100 for 30 min and washed again with PBS. After permeabilization, cells were incubated with the indicated primary antibodies, followed by appropriate fluorophore-conjugated secondary antibodies. GFP-KDEL was used as an ER marker, and nuclei were counterstained with 4′,6-diamidino-2-phenylindole for 2 min and washed three times with PBS. Images were acquired using a Zeiss confocal microscope (LSM 980).

### Biolayer interferometry

BLI was performed using a Gator Prime instrument (GatorBio) to measure the binding affinity between BiP and Omp25. Anti-GST biosensors (GatorBio) were used to immobilize GST-tagged BiP at 5 μg/ml for 300 s. Serial dilutions of His-Omp25 were prepared, starting from 7.69 μM with a 1.3-fold dilution across six concentrations. The assay protocol for each concentration included the following steps: initial baseline (60 s), BiP immobilization (300 s), second baseline (120 s), association phase (300 s), and dissociation phase (400 s). All binding assays were performed in PBS (pH 7.5) containing 0.02% Tween-20. Data were processed and plotted using GraphPad Prism (GraphPad Software, Inc). The *K*_*D*_ was calculated based on global fitting of the association and dissociation curves.

### Molecular docking

Structural docking of Omp25 onto BiP was performed using AlphaFold2 with default parameters (47, 48). A single Omp25 molecule was docked onto BiP, revealing that residue S105 of Omp25 was positioned near K447 of BiP (∼2.0 Å), and G108 was located near T445 of BiP (∼1.8 Å), suggesting potential hydrogen bond interactions between these residues.

### *Brucella* infection *in vitro*

RAW264.7 cells were seeded in 24-well plates at a density of 5 × 10^5^ cells per well and infected with *B. abortus* WT or Δ*omp25* strain of S2308 and A19. Following infection, cells were incubated with medium containing 50 μg/ml gentamicin for 1 h to eliminate extracellular bacteria and subsequently maintained in medium with 25 μg/ml gentamicin to prevent reinfection. For Tm pretreatment, cells were incubated with Tm for 30 min prior to infection (19).

To quantify intracellular bacterial loads, cells were lysed at the indicated time points using 0.5 ml PBS containing 0.5% Triton X-100. Lysates were serially diluted and plated on TSA to determine CFUs.

For analysis of gene and protein expression, infected cells were collected at different time points postinfection. Cell samples were harvested either in RNAiso Plus reagent for RNA extraction and RT–qPCR or in SDS sample buffer for immunoblot analysis.

### Mouse infection

BALB/c male mice (6–8 weeks old) were intraperitoneally infected with 3 × 10^5^ CFU of *B. abortus* S2308 WT or Δ*omp25* strain. At 20 days postinfection, bacterial burdens in the spleen were determined by plating tissue homogenates on TSA agar. Spleens were also weighed to assess splenomegaly and processed for histological examination using H&E staining.

For the pregnancy model, C57BL/6 pregnant mice (8 weeks old) were intraperitoneally injected with approximately 1.5 × 10^5^ CFU of *B. abortus* S2308 WT or Δ*omp25* strain at 4 days after timed-mating. Placentas were collected at 14 days postinfection to assess bacterial colonization and histopathological changes in the junctional and labyrinth zones.

### Statistical analysis

All experiments were performed with at least three independent biological replicates. Statistical analyses were conducted using GraphPad Prism software. Depending on the experimental design, unpaired *t* tests or one-way or two-way ANOVA were applied as appropriate. For immunoblot analyses, band intensities were quantified by using ImageJ softwase (NIH, Bethesda, MD) after normalization to the indicated loading controls. Statistical significance was defined as ∗*p* < 0.05 or ∗∗*p* < 0.01; ns, nonsignificant, as indicated in the figure legends.

## Data availability

All data are contained within the article.

## Supporting information

This article contains supporting information.

## Conflict of interest

The authors declare that they have no conflicts of interest with the contents of this article.
