# Supplementary material for: Brucella Omp25 activates the unfolded protein response to promote intracellular proliferation and inflammation
Source: J Biol Chem. 2026 Feb 26;302(4):111333. doi: 10.1016/j.jbc.2026.111333 (PMC13022623; doi:10.1016/j.jbc.2026.111333)
Supplement: Supporting information [file mmc1.pdf]

Supporting Information for:

Brucella Omp25 activates the unfolded protein response to promote intracellular proliferation and inflammation

Jin-Ke Yang[1,3], Shuang Huang[1,3], Yuan-Pan Hou[1,3], Hui-Fei Yuan[1,3], Yue-Wang[1,2,3], Mi Li[4], Li-Bo Cao[1,2,3], Tian Xia[1,3,*], Hong-Bing Shu[1,2,3,4,*], Xin Wu[1,3,*]

This file includes:

Table S1

Figure S1 to S5

Table S1

Gene	Forward	Reverse
qPCR-Human *BiP*	CTGTCCAGGCTGGTGTGCTCT	CTTGGTAGGCACCACTGTGTTC
qPCR-Human *CHOP*	GAACCAGGAAACGGAAACAG	ACCATTCGGTCAATCAGAGC
qPCR-Human *GADD34*	ACCTCTACTTCTGCCTTGTCTCC	TGGCTCCTTTACTTCTTTCTGTT
qPCR-Human *XBP1s*	CTGAGTCCGCAGCAGGTG	GAGATGTTCTGGAGGGGTGA
qPCR-Human *ERdj4*	TCTTAGGTGTGCCAAAATCGG	TGTCAGGGTGGTACTTCATGG
qPCR-Human *ATF4*	GGCCAAGCACTTCAAACATC	AAGCATCCTCCTTGCTGTTG
qPCR-Human *IL6*	TTCTCCACAAGCGCCTTCGGTC	TCTGTGTGGGGCGGCTACATCT
qPCR-Human *IKBA*	CGGGCTGAAGAAGGAGCGGC	ACGAGTCCCCGTCCTCGGTG
qPCR-Human *CXCL10*	GGTGAGAAGAGATGTCTGAATCC	GTCCATCCTTGGAAGCACTGCA
qPCR-Human *CXCL1*	CAGGGAATTCACCCCAAGAACA	GGATGCAGGATTGAGGCAAGC
qPCR-Human *GAPDH*	GTCTCCTCTGACTTCAACAGCG	ACCACCCTGTTGCTGTAGCCAA
qPCR-Mouse *Bip*	AGGATGCGGACATTGAAGAC	AGGTGAAGATTCCAATTACATTCG
qPCR-Mouse *Chop*	AGCCCTCTCCTGGTCTAC	AGGGAAGGATGAGGAAATCG
qPCR-Mouse *Gadd34*	GGCTGATAAGAGGCTTGGGG	CAGGGGTGCTGGGTTTGTAT
qPCR-Mouse *Il6*	TCTGCAAGAGACTTCCATCCAGTTGC	AGCCTCCGACTTGTGAAGTGGT
qPCR-Mouse *Il1b*	AAAGCCTCGTGCTGTCGGACC	CAGGGTGGGTGTGCCGTCTT
qPCR-Mouse *Tnfa*	GGTGATCGGTCCCCAAAGGGATGA	TGGTTTGCTACGACGTGGGCT
qPCR-Mouse *Gapdh*	ACGGCCGCATCTTCTTGTGCA	ACGGCCAAATCCGTTCACACC
RT-PCR-Human XBP1	ACAGCGCTTGGGGATGGATG	TGACTGGGTCCAAGTTGTCC
PCR-pUC19-*sacB*-LA-*omp25*	ACCTGCAGGCATGCAAGCTTAATGGCA ACCGGAAAACCATG	GTGTCCAATTATGCTATAAGGCATTCTC CTTACACAAATTACG
PCR-pUC19-*sacB*-*omp25*-RA	ATTTGTGTAAGGAGAATGCCTTATAGCA TAATTGGACACGGAAAACCG	ACCATGATTACGCCAAGCTTCCATCACG CAATATCTCAACCG
PCR-pUC19-*sacB*-LA-*omp25*-Flag	ACCTGCAGGCATGCAAGCTTATGCGCA CTCTTAAGTCTCTCGTA	CCAATTATGCTATAATTACTTGTCATCG TCGTCCTTGTAGTCGAACTTGTAGCCGA TGCC
PCR-pUC19-*sacB*-*omp25*-Flag- RA	CGGCATCGGCTACAAGTTCGACTACAA GGACGACGATGACAAGTAATTATAGCA TAATTGG	ACCATGATTACGCCAAGCTTGCTTTGCG ACGTTTTGCTGG
PCR-pBBR1MCS-2-*omp25*-Flag	GAAACAGTATTCAAGCTTATGCGCACTC TTAAGTCTCTCGT	ATCGATGATATCGAATTCTTACTTGTCA TCGTCGTCCTTGTAGTCGAACTTGTAGC CGATGCCG
PCR-pET-30c-*omp31b*	CGAGCTCCGTCGACAATGGCCGACATC ATCGTTGCTG	TCGAGTGCGGCCGCGAACTTGTAGTTCA GACCGAGGC
PCR-pET-30c-PERK^{LD}	CGAGCTCCGTCGACAATGTCAGCGACG CGAGTACCG	CTCGAGTGCGGCCGCATCCTTTTTGCGG ATATTCTTGTTGTAATG
PCR-pET-30c-IRE1α^{LD}	CGAGCTCCGTCGACAATGATTTTTGGAA GTACCAGCACAGTGAC	CTCGAGTGCGGCCGCGTCCTTAAGCATG GAGTCCACGG
PCR-pET-28a-sumo-ATF6^{LD}	CGAGCTCCGTCGACAATGGAACAGGAT TCCAGGAGAATGAACCC	CTCGAGTGCGGCCGCTTGTAATGACTCA GGGATGGTGCTG
PCR-pET-28a-sumo-*omp25*	GAGAACAGATTGGTGGATCCATGGCCG ACGCCATCCAGG	TCAGTGGTGGTGGTGGTGGTGCTCGAG GAACTTGTAGCCGATGCCG
PCR-pGEX-6p-1-BiP	AATTCCCGGGTCGACTCATGGAGGAGG ACAAGAAGGAGGACGT	CACGATGCGGCCGCCTACAACTCATCTT TTTCTGCTGTATCCTCT

Figure S1. PCR, RT-PCR and RT-qPCR primer sequences used in this study.

Figure S1

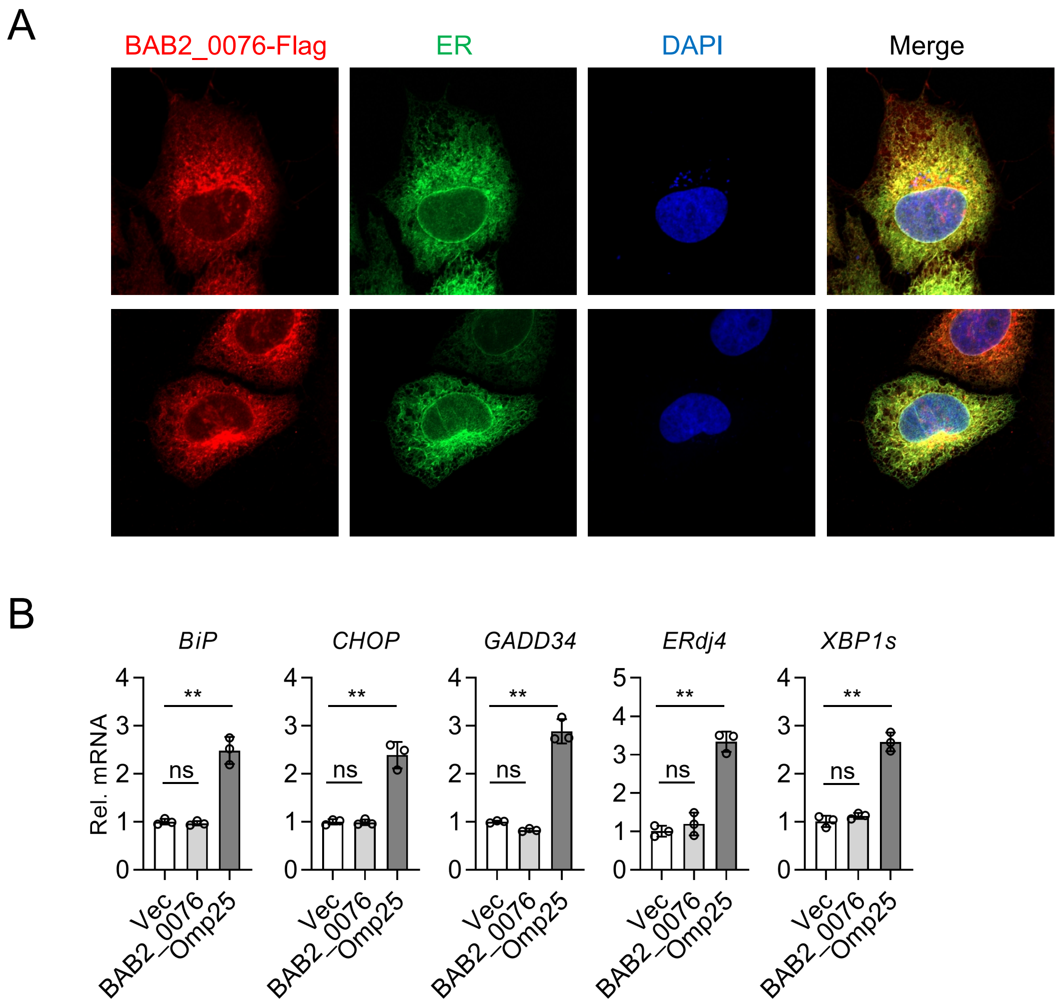

Figure S1. BAB2_0076 localizes to the ER but does not activate the UPR. (A) ER localization of BAB2_0076. HeLa cells (2×10^4) were co-transfected with BAB2_0076-Flag and an ER-GFP plasmid for 24 h, followed by immunostaining with an anti-Flag antibody and confocal microscopy. (B) Comparison of UPR gene induction by Omp25 and BAB2_0076. HEK293T cells (5×10^5) were transfected with empty vector, BAB2_0076-Flag (1 µg), or Omp25-Flag (1 µg) for 24 h, followed by RT-qPCR analysis of the indicated UPR-related genes. Data are presented as mean ± SD (n = 3 independent samples). Statistical analysis was performed using one-way ANOVA; **P < 0.01; ns, not significant.

Figure S2

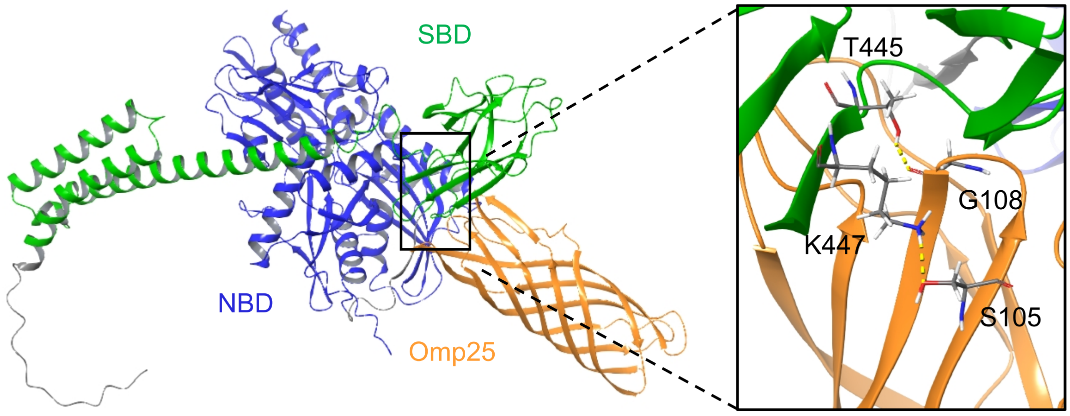

Figure S2. Predicted interaction between Omp25 and the substrate-binding domain (SBD) of BiP. Molecular docking model showing Omp25 (orange) bound to the SBD of BiP (green). Residues S105 and G108 of Omp25 are predicted to form hydrogen bonds with K447 and T445 of BiP, respectively (yellow dotted lines).

Figure S3

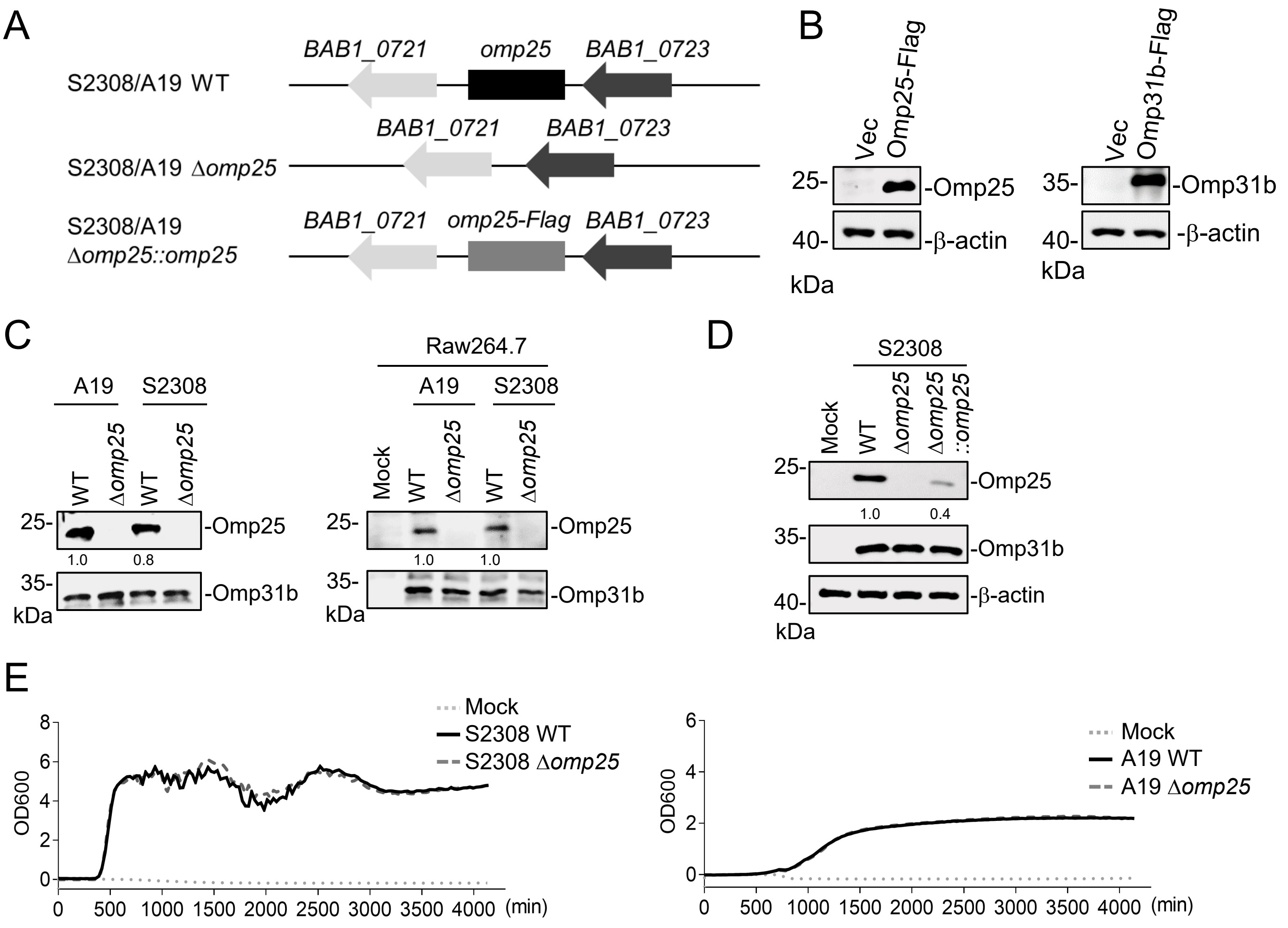

Figure S3. Generation and validation of *Brucella* Δ*omp25* and complementation strains. (A) Schematic diagram of the construction of Δ*omp25* and complemented (Δ*omp25::omp25*) strains. (B) Specificity of anti-Omp25 and anti-Omp31b antibodies. HEK293T cells were transfected with Omp25-Flag or Omp31b-Flag, and lysates were immunoblotted to confirm antibody specificity. (C) Validation of Δ*omp25* strains in S2308 and A19. Bacterial lysates from cultures and infected cells were analyzed by immunoblot analysis. Band intensities were quantified by ImageJ. (D) Validation of the Δ*omp25::omp25* complemented strain. RAW264.7 cells were infected with WT*, Δomp25*, or Δ*omp25::omp25* strain, and Omp25 expression was assessed by western blotting. (E) Growth kinetics of *B. abortus* WT and Δ*omp25* strains in TSB medium. Bacteria were cultured to mid-log phase (OD600 = 0.6), diluted 1:100 in fresh TSB, and OD600 was measured every 30 minutes over 72 hours.

Figure S4

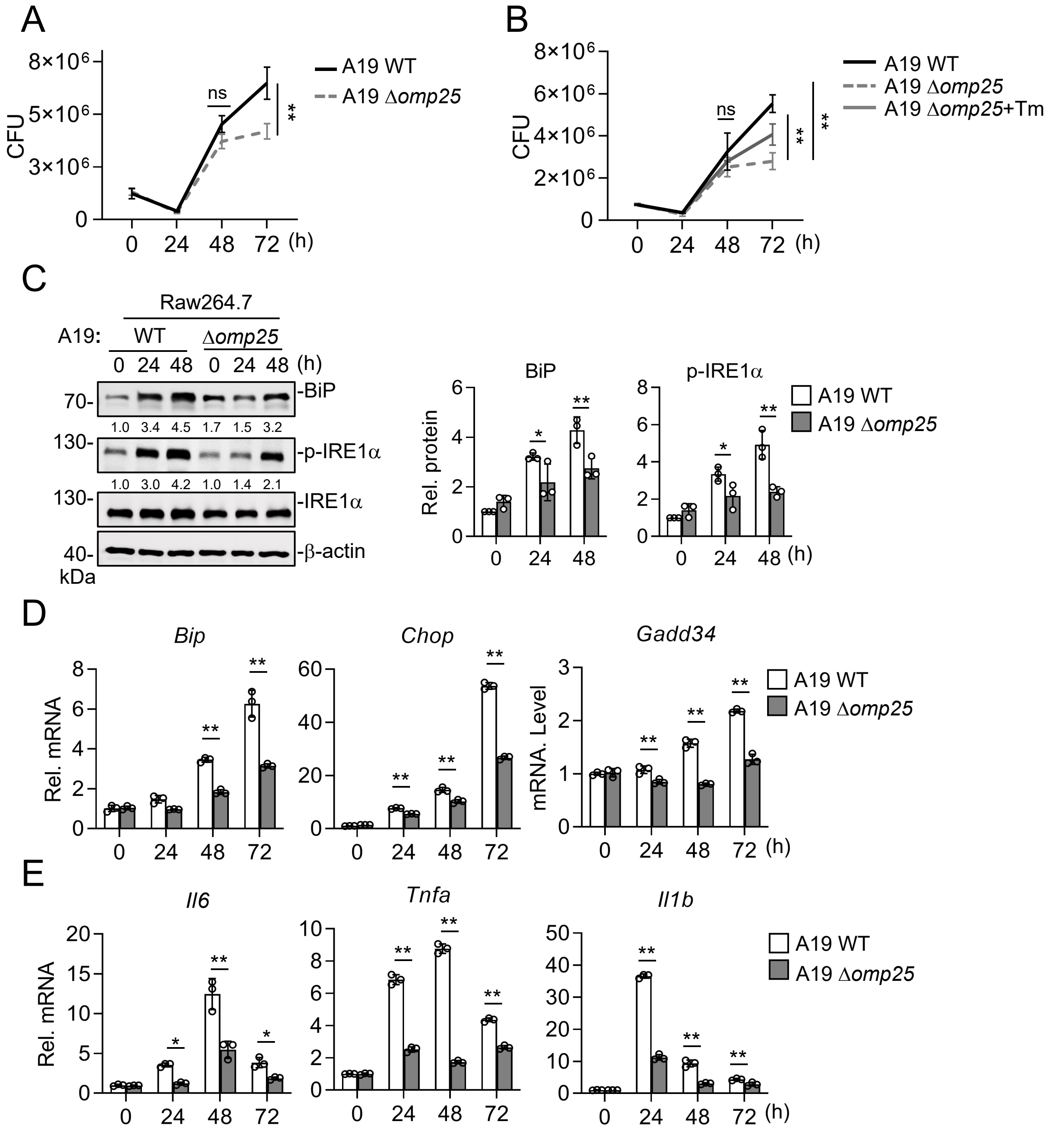

Figure S4. *omp25* deficiency attenuates *B. abortus* A19-induced UPR and inflammatory responses. (A) Intracellular replication of *B. abortus* A19 WT and Δ*omp25* strains. RAW264.7 cells (5×10^5) were infected at MOI = 200 for 4 h. After PBS washing, cells were incubated in DMEM with 1% FBS for 72 h. At indicated times, cells were lysed with 0.5% Triton X-100, and the number of viable intracellular bacteria were determined. (B) Effects of Tm pretreatment on Δ*omp25* intracellular replication. RAW264.7 cells (5×10^5) were pretreated with Tm (0.01 μg/mL) for 30 min before infection (MOI = 200), then processed as in (A) for CFU enumeration. (C) Effects of *omp25* deletion on *Brucella*-induced UPR. RAW264.7 cells (1×10^6) were left uninfected or infected (MOI = 200) with WT or Δ*omp25* strain. Lysates were analyzed by immunoblotting with the indicated antibodies. Band intensities were quantified by ImageJ. (D-E) Effects of *omp25* deletion on UPR signaling and inflammatory cytokine gene expression. RAW264.7 cells (5×10^5) were infected (MOI = 1000) with WT or Δ*omp25* strain for the indicated times, followed by RT-qPCR analysis. Data are mean ± SD (n = 3 independent samples). Statistical analysis was performed using two-way ANOVA; *$P < 0.05$, **$P < 0.01$, ns = not significant.

Figure S5

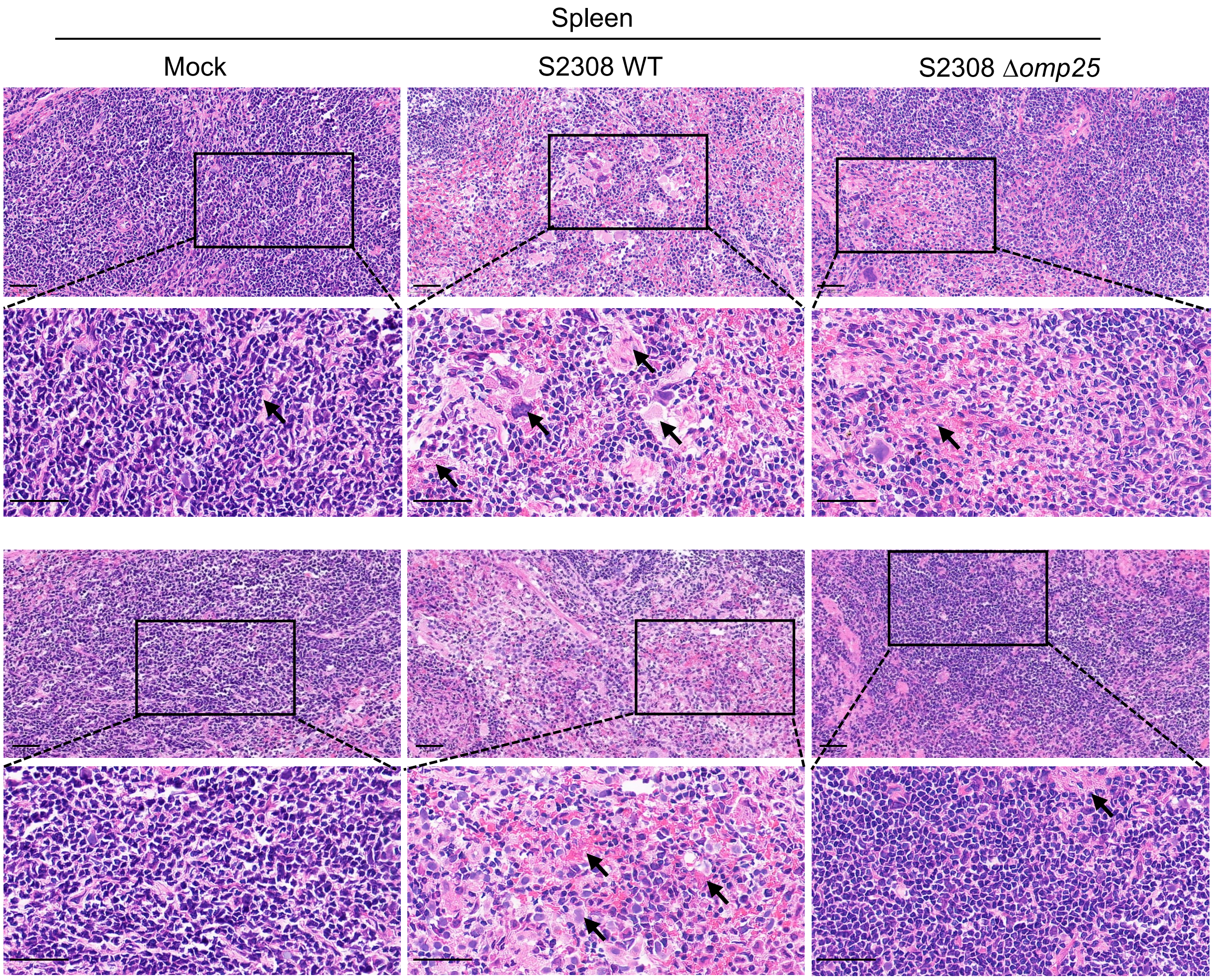

Figure S5. Histopathological analysis of spleens from *B. abortus* S2308-infected mice. Spleens were harvested from mice infected with S2308 WT or Δ*omp25* strain 20 days post-infection. Tissue sections were stained with H&E and examined by light microscopy. Scale bar, 50 μm.
